# Supplementary material for: Learning curve and surgical outcome of robotic assisted colorectal surgery with ERAS program
Source: Sci Rep. 2022 Nov 29;12:20566. doi: 10.1038/s41598-022-24665-w (PMC9709162; doi:10.1038/s41598-022-24665-w)
Supplement: Supplementary file 2 — Supplementary Information 2. [file 41598_2022_24665_MOESM2_ESM.zip › RAL Raw data-Ñ[▒K/127/HS_ñw╝╨░O▒Kñσ.pdf]

| 正式摘要(電子簽章：000000230323028) | □【讀取】憑證 |
| --- | --- |
| W55 / W55 □ 英文標題(注意:無英文姓名-請補病人基本檔!) | |

# 臺中榮民總醫院

## 出院病歷摘要

轉入號 : 轉出號 :

[redacted]

| | |
| --- | --- |
| (8)流水編號 | |
| (9)入院日期 | 110/11/17 (2021) 直腸外科 W55 - 241 |
| (10)轉科日期 | |
| (11)出院日期 | 110/11/22 (2021) 住院天數計 5 日 |

### 診斷

- 入院 :1. Lipoma over S-colon, with intussusception
- 出院 :1. Lipoma over S-colon, with intussusception, s/p Robotic assisted AR on 2021/11/18

### 主訴

- Intermittent abdominal pain

### 病史

- This is a 62 years old male patient with history of

  1. Lipoma over S-colon, with intussusception

  According to the patient, he had diagnosed of Lipoma over S-colon for years. Intermittent abdminal pain was noticed in recent days. Abd CT follow up showed 3.3cm Lipoma over S-colon with intussusception. Thus, he came to our CRS OPD for help. After discussion, he decided to received surgery intervention. Thus, this time he was admitted to our ward for operation.

### 體檢發現

- Anal inspection:
  Recto digital examination:
  Sigmoidoscopy:
  Anoscopy:
  Abdomen:
  - General appearance :
    - easy looking , conscious clear , without cardiopulmonary distress
    - Vital signs自動帶入date 體溫(BT) 脈搏(P) 呼吸(R) 血壓(BP) 身高(BH) 體重(BW) BMI
      20211117 36.6℃ 89次/分 18次/分 102/73mmHg 170cm 92kg 31.8
    - Vital signs: B.P. 102/73 mmHg , P.R. 89 /min , R.R. 18 /min , B.T. 36.6 ℃
  - HEENT :
    - Eyes: no anemic conjunctiva, no icteric sclera, normal EOM
    - ENT: no otorrhea, no rhinorrhea, no erythematous mucosa, no injected tonsil, no deformity
  - Neck: supple, freely movable, trachea midline,no deformity, no engorged jugular vein , no palpable thyroid or lymph node enlargement
  - Chest:
    - Inspection: Grossly normal, symmetric and fully expansion, no accessory muscle used, no operation scar, no spider angiomata, no gynecomastia
    - Palpation: no palpable mass or nodule, no subcutaneous emphysema, no tenderness
    - Ausculation: clear breathing sound
  - Heart:
    - Percussion: normal heart size
    - Ausculation: regular heart beat, normal S1, S2, no gallop, no murmur
  - Abdomen:
    - Inspection: in

- 
  - 
    - flat contour, symetrically, no scar, no stria, no dilated veins, no rashes
  - Ausculation: normoactive bowel sound, no abdominal bruits
  - palpation: soft, no muscle guarding or rigidity, no local tenderness, no rebounding pain, no palpable mass, no palpable pulsation
  - Percussion: no shifting dulllness, no hepatomegaly, no splenomagaly
- 

| Peripheral pulsation : | CA | BA | RA | FA | PA | PTA | DPA |
|---|---|---|---|---|---|---|---|
| Right side : | ++ | ++ | ++ | ++ | ++ | ++ | ++/+++ |
| Left side : | ++ | ++ | ++ | ++ | ++ | ++ | ++/+++ |

- Genito-anus: no tenderness or palpable mass lesion or rectal shelf, normal external genitalia, normal anal tone, brown stool

## CRS CONDITION

- 
  - Lung

## 手術日期及方法

- 手術日期及方法20211118 1. Robotic (XI) assisted anterior resection
  2. Intraoperative colonoscopy : (-).
  3. High ligation (-) : At IMA. Left colic artery and IMV were also ligated via robotic endoclips.
  4. Take down from the splenic flexure : (-)
  5. Robotic endoGIA (45-4.3) X2 + EEA (33mm) were applied
  6. Proximal cut end: >10 cm, distal cut end: 3 cm
  7. ADHESION BARRIER FILM was placed below the central abdominal wound

## 住院治療經過

- After admission, he received Robotic assist AR on 2021/11/18. He start Bland I diet on 11/19, and try bland III diet on 11/21. We add Econ, Through, Biofermin for him, and the wound pain tolerate after off PCA. Under relative stable condition, he was discharged on 11/22, OPD f/u.

## 合併症

## 一般檢查

- 累積報告 :

| DATE | TIME | ALB | TP | BIL.T | BIL.D | ALKP | AST | ALT | LDH | AMY | LIPASE | NH3 | Ferritin | UA |
|---|---|---|---|---|---|---|---|---|---|---|---|---|---|---|
| 1101117 | 2022 | 3.7 | | 0.75 | 0.17 | | 30 | 39 | | | | | | |
| 1101118 | 2239 | | | 0.9 | | | 30 | | | | | | | |

| DATE | TIME | WBC | RBC | HGB | HCT | MCV | PLT | BLST | NEUT | BAND | SEG | LYM | MON | EOS | BAS |
|---|---|---|---|---|---|---|---|---|---|---|---|---|---|---|---|
| 1101117 | 2019 | 6280 | | 15.8 | | | 157 | | 54.8 | | | 33.8 | 7.2 | 3.2 | 1.0 |
| 1101118 | 2239 | 13500 | | 15.8 | 46.8 | | 156 | | 92.2 | | | 6.4 | 1.3 | 0.0 | 0.1 |

| DATE | TIME | NA | K | CL | BUN | CREAT | eGFR | CA | Mg | P | CRP | ESR | PCT | D-Dimer | LACTATE |
|---|---|---|---|---|---|---|---|---|---|---|---|---|---|---|---|
| 1101117 | 2022 | 138 | 4.0 | | 15 | 1.04 | 76.91 | 8.9 | | | | | | | |
| 1101118 | 2239 | 140 | 4.1 | | 15 | 1.17 | 67.14 | 8.6 | | | | | | | 14.0 |

| DATE | TIME | GLUCOSE | HbA1C | CHOL | TG | HDL | LDL | KETONE | CK | CKMB | TRO-T | PT-P | APTT-P | NT-pro BNP |
|---|---|---|---|---|---|---|---|---|---|---|---|---|---|---|
| 1101117 | 2018 | | | | | | | | | | | 10.8 | 29.9 | |

## 特殊檢查

## 病理報告

## 其它

## 出院時情況

- 3.改門診治療

## 出院指示

- 

| 藥名 | 劑量 | 劑量單位 | 用法 | 頻次 | 數量 | 數量單位 | 現況 | 天數 | 單位 |
|---|---|---|---|---|---|---|---|---|---|
| $Biofermin tab | 2 | TAB | PO | TID | 48 | TAB | TH | 8 | 天 |
| Acetal tab 500mg | 1 | TAB | PO | Q6HPRN | 32 | TAB | TH | 8 | 天 |
| (□)Takepron OD tab 30mg | 1 | TAB | PO | QDAC | 8 | TAB | TH | 8 | 天 |
| Mag. Oxide tab | 2 | TAB | PO | TID | 48 | TAB | TH | 8 | 天 |
| | | | | | | | | | |

| | | | | | | | | | |
|---|---|---|---|---|---|---|---|---|---|
| Celebrex cap 200mg | 1 | CAP | PO | QD | 8 | CAP | TH | 8 | 天 |
| Through tab | 1 | TAB | PO | QN | 8 | TAB | TH | 8 | 天 |
| Econ tab 40mg | 1 | TAB | PO | QID | 32 | TAB | TH | 8 | 天 |
| OCAS Harnalidge 0.4 mg | 1 | TAB | PO | QDAC | 8 | TAB | TH | 8 | 天 |

## 出院計劃

- 門診日期
  - date on 2021/11/30

主治醫師 2062F 蔣鋒帆 / 住院醫師 2339C 蔡濬樟

正式摘要(電子簽章：**000000230323028)**

W55 / W55

doctorRank:B
